# Cervical medullary syndrome secondary to craniocervical instability and ventral brainstem compression in hereditary hypermobility connective tissue disorders: 5-year follow-up after craniocervical reduction, fusion, and stabilization

**DOI:** 10.1007/s10143-018-01070-4

**Published:** 2019-01-09

**Authors:** Fraser C. Henderson, C. A. Francomano, M. Koby, K. Tuchman, J. Adcock, S. Patel

**Affiliations:** 1 Doctor’s Community Hospital, Lanham, MD USA; 2 The Metropolitan Neurosurgery Group, LLC, Silver Spring, MD USA; 3 Harvey Institute of Human Genetics, Greater Baltimore Medical Center, Baltimore, MD USA; 4 Medical University of South Carolina, Charleston, SC USA

**Keywords:** Ehlers-Danlos syndrome, Craniocervical instability, Clivo-axial angle, Cervical medullary syndrome

## Abstract

A great deal of literature has drawn attention to the “complex Chiari,” wherein the presence of instability or ventral brainstem compression prompts consideration for addressing both concerns at the time of surgery. This report addresses the clinical and radiological features and surgical outcomes in a consecutive series of subjects with hereditary connective tissue disorders (HCTD) and Chiari malformation. In 2011 and 2012, 22 consecutive patients with cervical medullary syndrome and geneticist-confirmed hereditary connective tissue disorder (HCTD), with Chiari malformation (type 1 or 0) and kyphotic clivo-axial angle (CXA) enrolled in the IRB-approved study (IRB# 10-036-06: GBMC). Two subjects were excluded on the basis of previous cranio-spinal fusion or unrelated medical issues. Symptoms, patient satisfaction, and work status were assessed by a third-party questionnaire, pain by visual analog scale (0–10/10), neurologic exams by neurosurgeon, function by Karnofsky performance scale (KPS). Pre- and post-operative radiological measurements of clivo-axial angle (CXA), the Grabb-Mapstone-Oakes measurement, and Harris measurements were made independently by neuroradiologist, with pre- and post-operative imaging (MRI and CT), 10/20 with weight-bearing, flexion, and extension MRI. All subjects underwent open reduction, stabilization occiput to C2, and fusion with rib autograft. There was 100% follow-up (*20/20*) at 2 and 5 years. Patients were satisfied with the surgery and would do it again given the same circumstances (100%). Statistically significant improvement was seen with headache (8.2/10 pre-op to 4.5/10 post-op, *p <* 0.001, vertigo (92%), imbalance (82%), dysarthria (80%), dizziness (70%), memory problems (69%), walking problems (69%), function (KPS) (*p <* 0.001). Neurological deficits improved in all subjects. The CXA average improved from 127° to 148° (*p <* 0.001). The Grabb-Oakes and Harris measurements returned to normal. Fusion occurred in 100%. There were no significant differences between the 2- and 5-year period. Two patients returned to surgery for a superficial wound infections, and two required transfusion. All patients who had rib harvests had pain related that procedure (3/10), which abated by 5 years. The results support the literature, that open reduction of the kyphotic CXA to lessen ventral brainstem deformity, and fusion/stabilization to restore stability in patients with HCTD is feasible, associated with a low surgical morbidity, and results in enduring improvement in pain and function. Rib harvest resulted in pain for several years in almost all subjects.

## Introduction

Many studies have drawn attention to the presence of craniocervical instability or basilar invagination in patients with Chiari one and Chiari zero malformation [1–23]. The need for reduction and stabilization in basilar invagination and craniocervical instability are recognized in connective tissue joint degenerative disorders, such as rheumatoid arthritis and lupus [10, 17, 24–36] and hereditary hypermobile and developmental disorders, including osteogenesis imperfecta, achondroplasia, Down syndrome and Ehlers-Danlos syndrome (EDS) [8, 18, 21, 26, 31, 37–50].

Emblematic of the approximately 50 heritable connective tissue disorders characterized by joint hypermobility is Ehlers-Danlos syndrome (EDS). Though Ehlers-Danlos syndrome was described in 1905, its neurological and spinal manifestations have only recently been appreciated [18, 41, 51–66]. These heritable connective tissue disorders are characterized by tissue fragility, skin extensibility, joint hypermobility, premature disk degeneration and spinal problems, and numerous comorbid conditions.

We report on an IRB-approved retrospective cohort study of 20 consecutive patients with hereditary connective tissue disorders and a kyphotic CXA, cerebellar ectopia (18/20), and craniocervical instability or ventral brainstem compression, who underwent reduction and stabilization. This is the first such study to critically assess 5-year outcomes after craniocervical reduction, stabilization, and fusion in a patient population with hereditary connective tissue disorders.

In this study, the CXA (clivo-axial angle) was used to indicate *potential* brainstem deformity. The CXA has drawn increasing attention as an important radiological metric to indicate the presence of neurological deficit and consideration for craniocervical stabilization [4]. The line of reasoning that a kyphotic CXA is associated with pathologic bending of the brainstem (medullary kyphosis, or kink) began with Liszt, who first recognized that clivo-axial kyphosis may result in neurobehavioral effects. Van Gilder reported that CXA of less than 150° were often associated with neurological deficits [67]. Breig demonstrated the importance of mechanical tension and deformation of the brainstem [68]. Menezes described the “fulcrum effect in basilar invagination, by which traction is applied to the caudal brainstem and rostral cervical spinal cord. Others have demonstrated the salutary consequences to the correction of the CXA [1, 10, 12, 15, 30, 49, 69–75].

It is important to recognize that the CXA is simply a static representation of a dynamic phenomenon. It has been generally considered that a CXA of less than 135° represents the threshold below which chronic repetitive injury may occur as a result of mechanical deformation of the lower brainstem and upper spinal cord.

The authors’ hypothesis was that reduction of the Clivo-axial kyphosis and stabilization for craniocervical instability were feasible and associated with clinical improvement in the hereditary connective tissue disorder (HCTD) population.

## Materials and methods

### Subject enrollment

Over a 2-year period (2011–2012), a cohort of 22 consecutive patients diagnosed with EDS, or in a few cases, unspecified hereditary connective tissue disorders (HCTD), were enrolled in the study and underwent occipital to C1/C2 fusion for craniovertebral instability and flexion deformity. Of the original 22 consecutive subjects, two were excluded: one had previously undergone a cranio-spinal fusion, and the second declined to participate due to unrelated medical issues. The data analysis was, therefore, conducted on the remaining 20 subjects, all of whom were enrolled in the IRB-approved study **(**IRB# 10-036-06: Greater Baltimore Medical Center). In 18 patients, cerebellar ectopia was also present.

### Evaluation

**Fig. 1:**
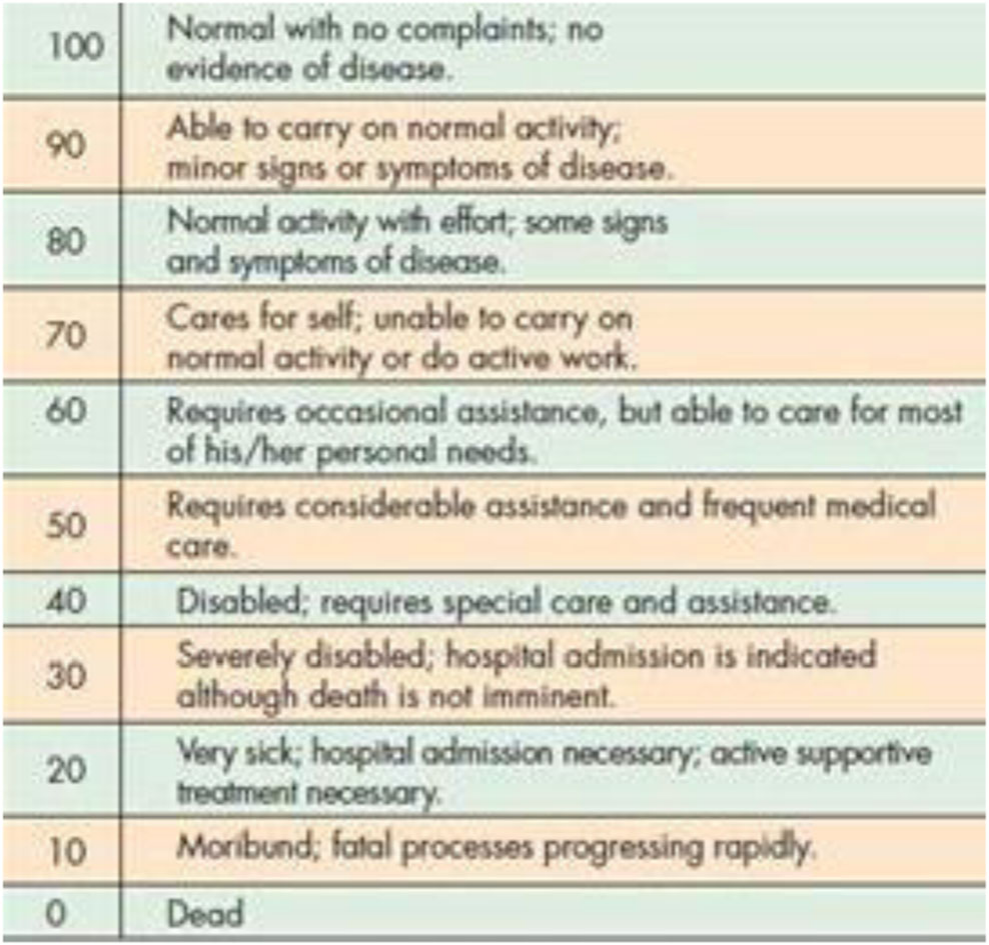
The Karnofsky Performance Status Scale

Symptoms were assessed by a standardized questionnaire administered by third party at 2 and 5 years. Pain was assessed by the visual analog scale for pain (0–10/10). The neurologic exams were performed by the neurosurgeon. Function and the ability to return to work were assessed with the Karnofsky Performance Scale (Fig. 1). Radiological measurements were performed by a neuroradiologist (MK) after 2 years.

Pre- and post-operative radiological measurements were made or reviewed by the neuroradiologist (MK). Subjects underwent pre-operative and post-operative imaging with MRI and CT of the cervical spine. Upright, weight-bearing flexion and extension MRI of the cervical spine was obtained in 10/20 of the subjects.

**Fig. 2:**
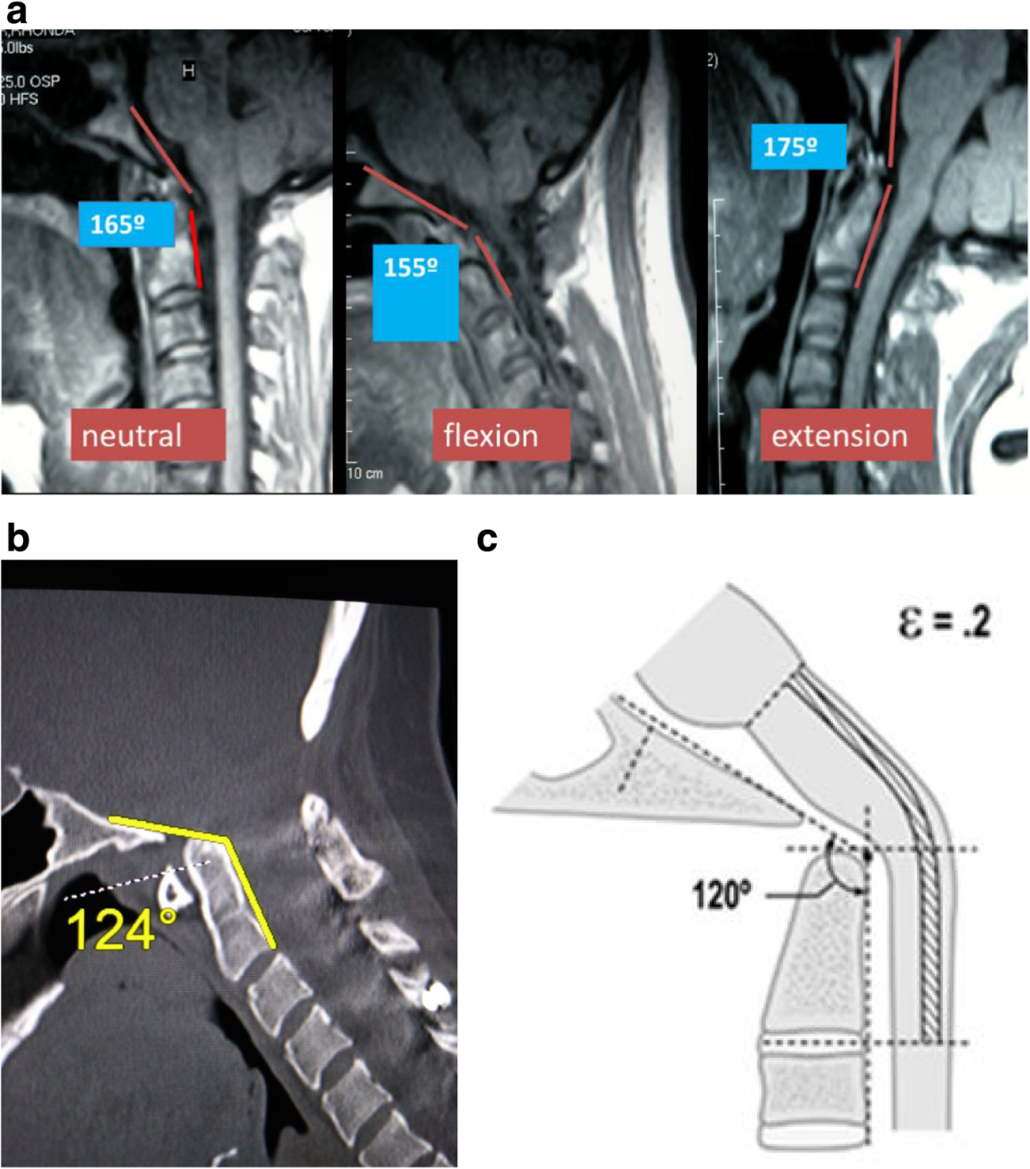
**a** The normal CXA. The normal CXA is approximately 155°, decreasing 10° in flexion and increasing 10° in extension. **b** The pathological clival axial angle (CXA) is more kyphotic than the normal CXA. The CXA is subtended by the posterior axial line and a line drawn along the surface of the lower third of the clivus. An angle of 135° or less is considered potentially pathological. The kyphotic CXA of 124° shown here is clearly pathological and results in a mechanical deformity and lengthening of the brainstem and upper spinal cord, as shown diagrammatically in the next image (Fig. 2c). **c** Diagrammatical rendering of a kyphotic CXA. In hereditary connective tissue disorders, ligamentous laxity may thus result in a kyphotic CXA in flexion, with a concurrent increase in strain (Ɛ)

Radiometrics were performed at the 2-year follow-up and included the clivo-axial angle (CXA), Grabb-Mapstone-Oaks measurement (the pBC2), and the horizontal Harris Measurement (Basion axis interval or BAI). CXA is the measurement in degrees between the line drawn along the lower third of the clivus, and a line drawn along the posterior aspect of the axis [1, 76] (Fig. 2a). The CXA measurements were taken from the flexion image, when it was available (Fig. 2b, c**)**.

**Fig. 3:**
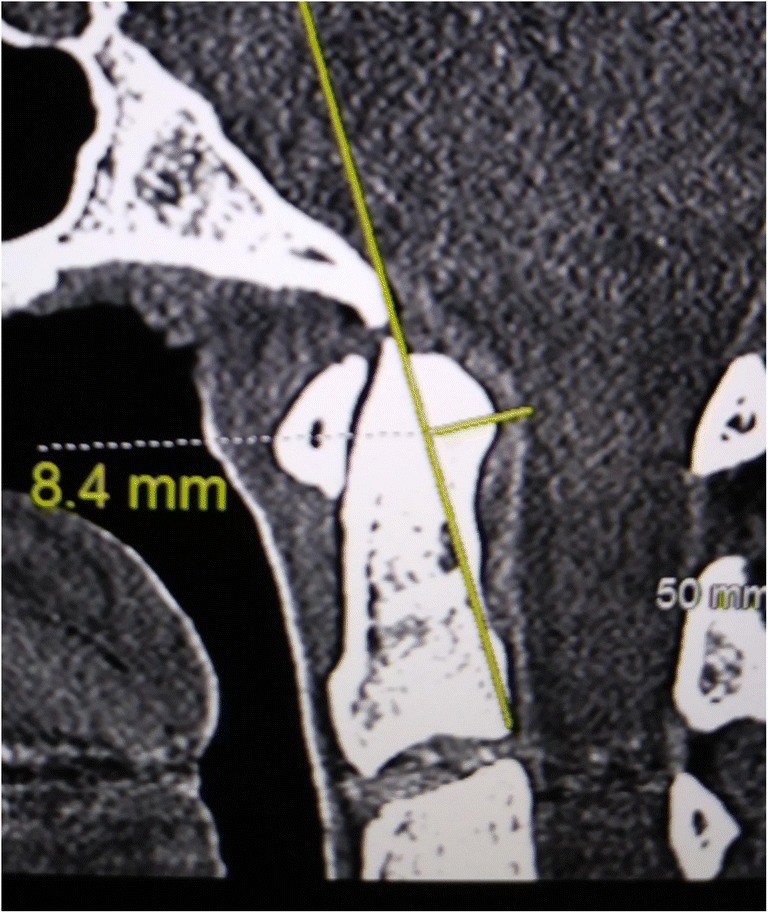
The Grabb, Mapstone, Oakes measurement: a measurement of 9 mm or greater implies a high risk of ventral brainstem compression

The *pBC2*, or *Grabb*, *Oakes measurement* (Fig. 3) is the perpendicular measurement from the dura to a line drawn from the basion to the posterior inferior aspect of C2 [7, 76, 77].

**Fig. 4:**
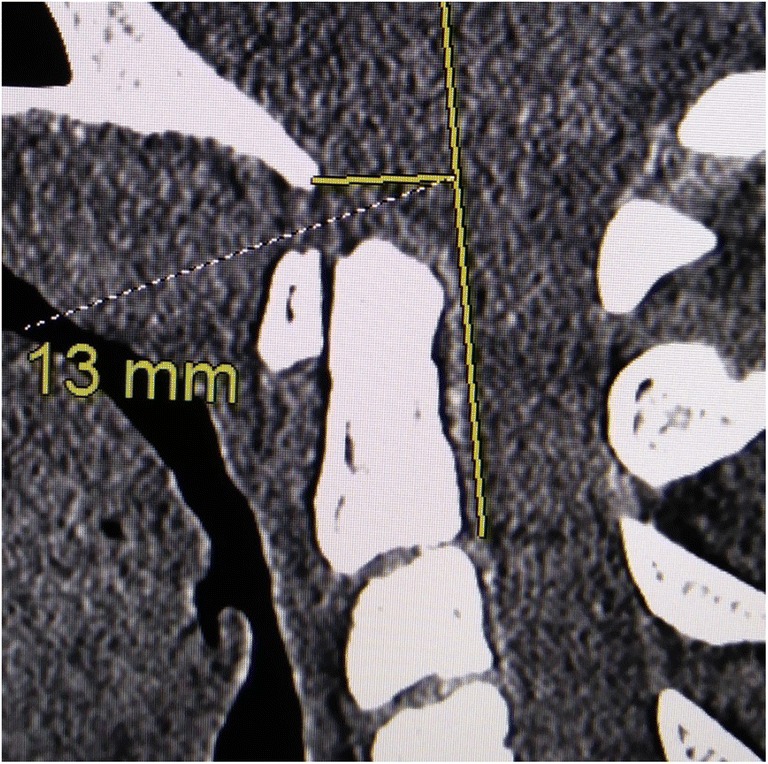
Horizontal Harris Measurement (HHM): a measurement of > 12 mm represents craniocervical instability. If the HHM changes by > 2 mm between flexion and extension, then craniocervical instability is inferred

*Horizontal Harris measurement* or *BAI* is the distance from the basion drawn perpendicularly to the posterior axial line (PAL) (Fig. 4). A measurement greater than 12 mm represents instability [76–78]. When possible, the Harris measurement/BAI is made from the MRI or CT in both flexion and extension to assess translation (sliding movement) between flexion and extension.

### Inclusion criteria for occipital-cervical fusion stabilization surgery

All subjects met the following criteria:

i. Formal genetics evaluation and diagnosis with a hereditary connective tissue disorder (CF)
ii. Signed consent
iii. Severe headache and/or neck pain greater than or equal to 7/10 by the visual analog scale for greater than 6 months.
iv. Symptoms of the cervical medullary syndrome [1, 79]
v. Demonstrable neurological deficits
vi. Congruent radiological findings were in accordance with the treatment algorithm previously set forward [70], including kyphotic CXA (less than 135°), craniocervical instability (greater than Harris/BAI measurement of 4 mm*), or low-lying cerebellar tonsils or Chiari malformation.
vii. Failed conservative treatment (physical therapy, activity modification, pain medications, neck brace, and in some circumstances, chiropractic, electrical stimulation, massage)

*Note: The normal Harris/BAI measurement changes no more than 1 mm between flexion and extension. The authors allowed 3 mm for error.

#### Operative technique

Preoperative traction reduction was not performed*.* Subjects were intubated in the neck brace with a GlideScope intubation technique to improve the view of the glottis and to avoid hyperextension of the neck. Sensory evoked potentials were performed throughout the surgery. A three-pronged Mayfield head holder was placed, and the subject positioned prone on chest rolls. The cervical spine was carefully aligned to eliminate tilt and rotation, and then placed in a neutral position, as confirmed by cross table fluoroscopy. After sterile prep and drape, the incision was made from inion to C4, but the subperiosteal exposure was limited to the occiput, C1and C2. Care was taken to preserve the ligaments attached to the dorsal aspect of the spinous process of C2 and to the caudal aspect of the C2 lamina.

A limited sub occipital decompression was performed with high speed burr and Kerrison rongeur from the foramen magnum upward 14 mm, but carried laterally to the full meridian of the dura. The dura was not opened, and thus, no expansion duroplasty was performed.

**Fig. 5:**
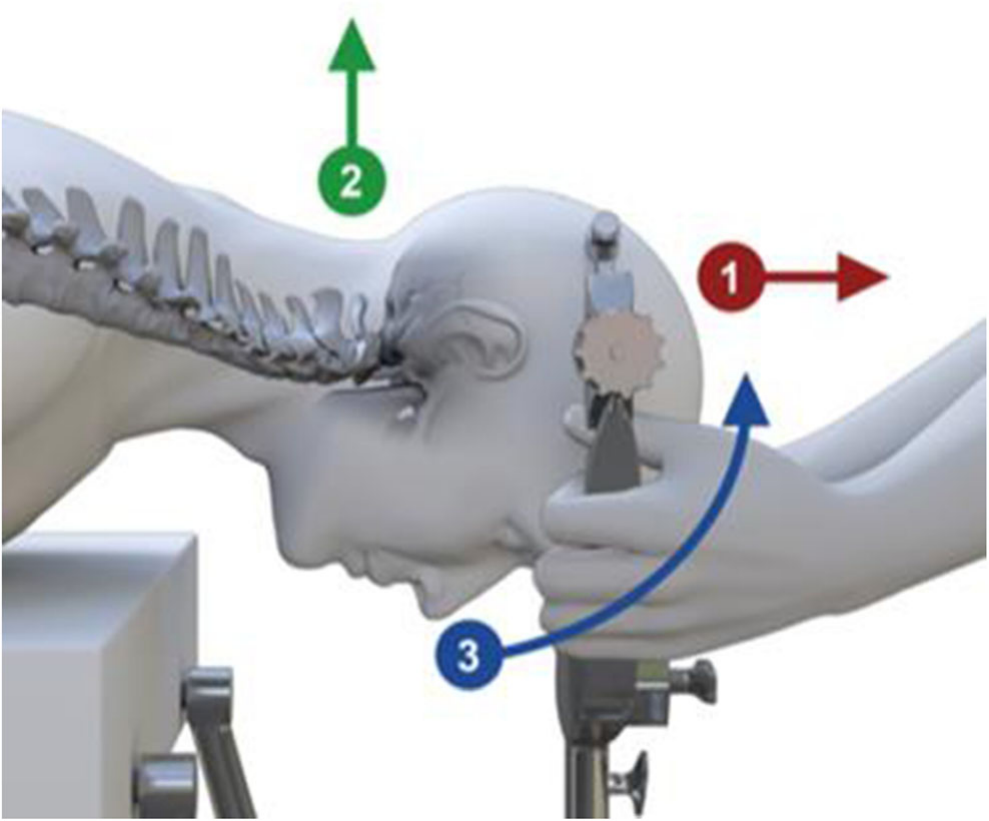
Traction reduction: the surgeon stands at the head of the table, grasps the head holder, and applies 1: traction; 2: posterior translation; 3: extension, to bring the basion into correct relationship with the odontoid

**Fig. 6:**
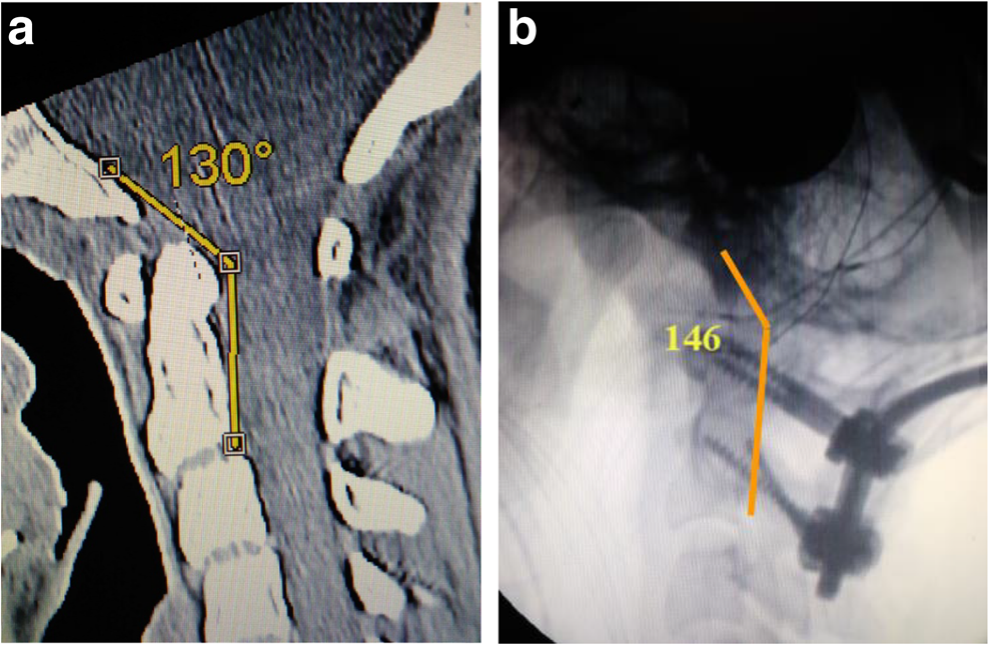
Intraoperative reduction: the preoperative CT (i) shows a CXA of 130°; the intra-operative fluoroscopic image after reduction (ii) shows a CXA of 146°

Open reduction of the craniocervical junction was performed to normalize the CXA. To accomplish the open reduction, the surgeon stepped to the head of the table, applied traction, posterior translation, and extension at the craniocervical junction. The head holder was then locked in place and checked with fluoroscopy (Fig. 5). Sensory and motor-evoked potentials were continuously monitored throughout the procedure. The reduction was accomplished in one to four iterations, under fluoroscopic guidance, with the goal of increasing the CXA by approximately 20° [10, 12, 70] and to bring the basion over the midpoint or anterior half of the odontoid **(**Fig. 6**)**.

These subjects underwent a craniocervical fusion and stabilization in order to maintain the corrected CXA and relationship of the basion to the odontoid process and to stabilize the craniocervical junction. To accomplish the stabilization, a titanium plate (Nex-Link OCT® Occipital cervical plating system, Zimmer) was contoured slightly and affixed to the occiput. Titanium 3.5-mm screws were placed in the C1 lateral masses and the C2 pedicles bilaterally. After reduction, the screws were connected by rods to the occipital plate [80–82]. In one case, it was necessary to place screws in the C3 lateral masses to achieve adequate stability.

To accomplish the fusion, bone surfaces were decorticated. Two rib autografts were harvested at approximately the T7 level [83]. The rib grafts were contoured to fit from the suboccipital bone to the upper cervical vertebrae, augmented with demineralized bone matrix, and secured with number one proline to prevent migration of the graft.

Both the neck and graft harvest wounds were then closed over drains. The patients were usually mobilized 1 day after surgery and kept in a neck brace (Miami J™, or equivalent) for 4 weeks. Physical therapy was then started.

#### Statement of human and animal rights

All procedures performed in studies involving human participants were carried out in accordance with the ethical standards of the institutional and/or national research committee in the United States, and with the 1964 Helsinki declaration and its later amendments or comparable ethical standards. Informed consent was obtained from all individual patients and participants included in the study.

## Results

Nineteen subjects were female and one male, with an average age of 24 years (range of 12–53 years). All patients were diagnosed with a hereditary connective tissue disorder (HCTD): ten had hypermobile EDS (h-EDS), two classical EDS, four unspecified EDS, and four hypermobility spectrum disorder. All subjects (20/20) had a kyphotic CXA (less than or equal to 135°) and craniocervical instability (Harris Measurement/BAI of 4 mm or greater). Eighteen subjects had cerebellar ectopia.

### Pre-operative findings

The most prominent symptoms prior to surgery included headache (100%), fatigue (100%), dizziness (100%), muscle pain, vertigo, arm weakness, neck pain, balance problems, memory problems, night awakenings, numbness and weakness of the arms and legs, and gait problems (Table 1**).**

**Table 1:** Two-year follow-up: presence and change in frequency of symptoms/problems among participants (*n* = 20)

Symptom/problem	% Pre-surgery	% Post-surgery	%With improvement in frequency post-surgery^a^	% With worsening of frequency post-surgery^a^	*p* value^b^	% With onset post-surgery^c^
*Headaches*	100%	95% (19/20)	85% (17/20)	0	*< 0.001*	0
Fatigue	100%	100%	30% (6/20)	15% (3/20)	NS	0
*Dizziness*	100%	95% (19/20)	70% (14/20)	10% (2/20)	*< 0.0007*	0
Muscle pain	95% (19/20)	95% (19/20)	36.8% (7/19)	10.5% (2/19)	NS	0
Upper extremity weakness	90% (18/20)	85% (17/20)	61.1% (11/18)	22.2% (4/18)	NS	0
Joint pain	85% (17/20)	85% (17/20)	29.4% (5/17)	11.8% (2/17)	NS	0
Neck pain	85% (17/20)	90% (18/20)	70.6% (12/17)	5.9% (1/17)	NS	33.3% (1/3)
*Balance problems*	85% (17/20)	85% (17/20)	82.4% (14/17)	5.9% (1/17)	*< 0.0001*	0
Night awakenings	85% (17/20)	85% (17/20)	23.5% (4/17)	11.8% (2/17)	NS	0
*Memory problems*	80% (16/20)	80% (16/20)	68.9% (11/16)	0	*< 0.002*	0
*Walking problems*	80% (16/20)	70% (14/20)	68.9% (11/16)	6.3% (1/16)	*< 0.002*	0
Upper extremity numbness	75% (15/20)	85% (17/20)	73.3% (11/15)	6.7% (1/15)	NS	40% (2/5)
Hands and feet turning cold	75% (15/20)	70% (14/20)	26.75% (4/15)	6.7% (1/15)	NS	0
Lower extremity numbness	75% (15/20)	70% (14/20)	60% (9/15)	13.3% (2/15)	NS	0
Visual problems	75% (15/20)	80% (16/20)	53.3% (8/15)	13.3% (2/15)	NS	20% (1/5)
Lower extremity weakness	65% (13/20)	70% (14/20)	69.2% (9/13)	15.4% (2/13)	NS	14.3% (1/7)
*Vertigo*	65% (13/20)	50% (10/20)	92.3% (12/13)	0	*< 0.0006*	0
Hearing problems	65% (13/20)	65% (13/20)	61.5% (8/13)	15.4% (2/13)	NS (0.053)	14.3% (1/7)
*Speech problems*	60% (12/20)	55% (11/20)	80% (8/12)	8.3% (1/12)	*< 0.03*	0
*Frequent daytime urination (>every 2 h)*	60% (12/20)	45% (9/20)	41.7% (5/12)	0	*< 0.02*	0
GERD	55% (11/20)	55% (11/20)	36.4% (4/11)	0	NS	11.1% (1/9)
Swallowing/choking problems	55% (11/20)	55% (11/20)	63.4% (7/11)	18.2% (2/11)	NS	22.2% (2/9)
Nocturia (> twice a night)	55% (11/20)	55% (11/20)	27.3% (3/11)	9.1% (1/11)	NS	11.1% (1/9)
IBS	50% (10/20)	50% (10/20)	30% (3/10)	0	NS	0
Tremors	40% (8/20)	40% (8/20)	87.5% (7/8)	0	NS	0
Fainting	35% (7/20)	25% (5/20)	85.7% (6/7)	0	NS	14.3% (1/7)
Numbness in back	30% (6/20)	40% (8/20)	66.7% (4/6)	0	NS	14.3% (2/14)
Sleep apnea	25% (5/20)	25% (5/20)	20% (1/5)	0	NS	0

^a^For those participants who had presence of symptom/problem prior to surgery

^b^Comparing frequencies of symptom/problem pre vs. post-surgery, a significant *p* value indicates less frequent symptom/problem post-surgery

^c^For those participants who did not have the presence of symptom/problem prior to surgery

### Patient satisfaction

**Fig. 7:**
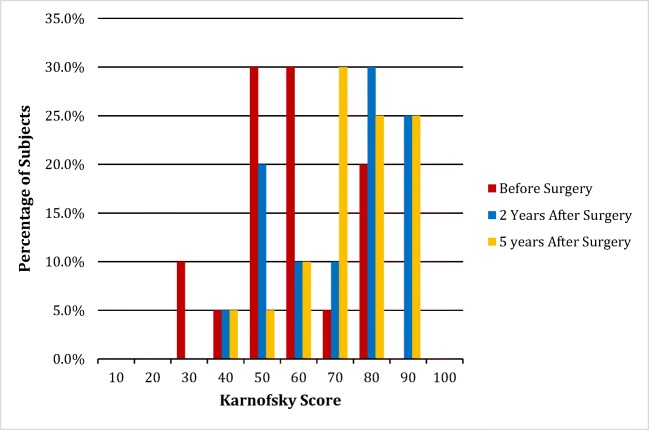
Comparison of Karnofsky scores before surgery and at 2 and 5 years post-surgery

**Fig. 8:**
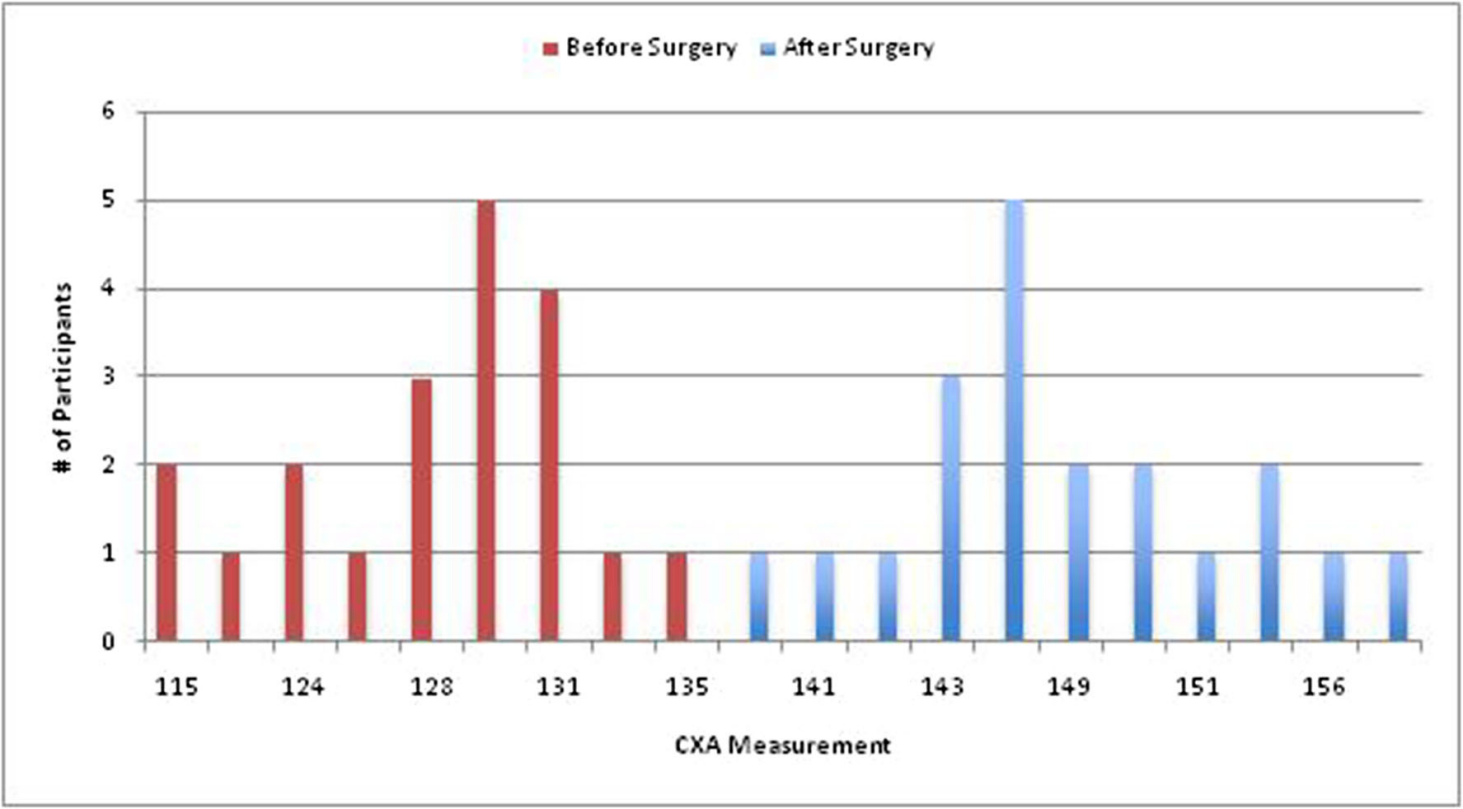
Comparison of CXA measurements pre vs. post-surgery

**Fig. 9:**
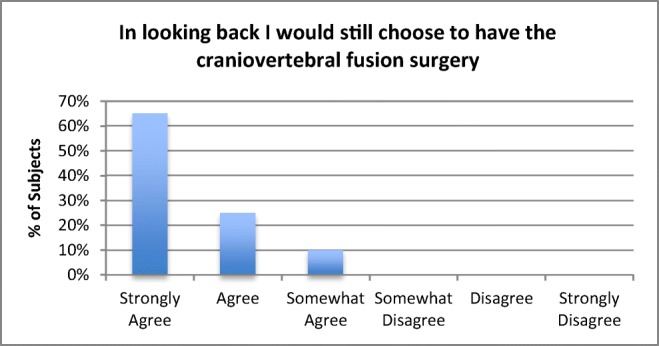
Opinion regarding choice of surgery

**Fig. 10:**
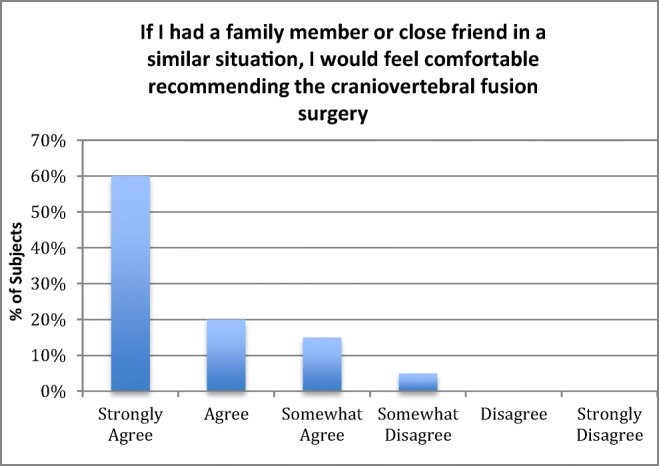
Opinion regarding recommending surgery

**Fig. 11:**
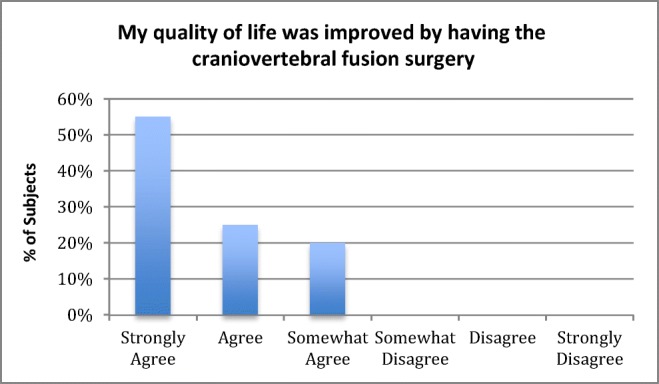
Opinion regarding improvement of quality of life

**Fig. 12:**
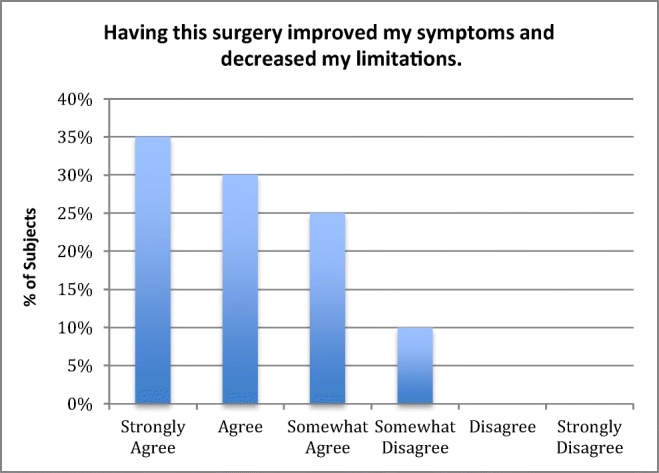
Opinion regarding symptoms and limitations

There was 100% follow-up at 2 years and 5 years (Figs. 7 and 8). All patients were satisfied with the surgery and would repeat the surgery given similar circumstances, and reported improved quality of life (Figs. 9, 10, and 11). All but one patient would recommend the surgery to a family member (Fig. 10). Eighteen of the twenty patients reported that the craniocervical fusion surgery had decreased their limitations; the remaining two patients, who responded that the limitations had not decreased with surgery, explained that there remained limitations from other medical problems and spinal instability elsewhere (Fig. 12).

### Postoperative findings

Postoperatively at 2 years, statistically significant improvements were seen in vertigo (92%), headaches (85%), imbalance (82%), dysarthria (80%), dizziness (70%), memory (69%), walking (69%), and frequent daytime urination (42%) (Table 1). The average headache decreased from 8.1/10 pre-op to 4.35/10 post-op (*p <* 0.0001). Neck pain mean decreased in 71% of patients, from 6.45/10 to 4.05/10 post-op (*p <* 0.002), and muscle pain decreased from 6/10 to 4.7/10 post-op (*p <* 0.009) (Table 2**)**.

**Table 2:** Two- and five-year follow-up: comparison of pain levels (0–10 scale) among participants pre- vs. post-surgery (*n* = 20)

Area of pain	Pre-surgery	2 year post-surgery	*p* value	5 year post-surgery	*p* value
***Headaches***	8.10	4.35	*< 0.0001*	5.75	*< 0.00002*
***Neck***	6.45	4.05	*< 0.002*	4.7	*NS (< .091)*
Joints	5.30	4.60	NS	3.70	*<* 0.018
*Muscles*	5.95	4.70	*< 0.009*	*4.45*	*NS (< .069)*

Improvement, though not statistically significant, included tremors (87%), syncope (86%), numbness of the arms and hands (73%), upper extremity numbness (73%), lower extremity weakness (69%), back numbness (67%), swallowing difficulty (63%), upper extremity weakness (61%), hearing problems (61%), lower extremity numbness (55%), and GERDS (55%) (Table 1).

Similarly, at 5 years, there remained statistically significant improvement in dizziness (75%), walking problems (69%), speech problems (67%), frequent daytime urination (67%), headaches (65%), and imbalance (59%)**.** Improvement in upper extremity numbness, syncope, lower extremity weakness, back numbness, swallowing difficulty, upper extremity weakness, hearing problems, and lower extremity numbness were improved but not with statistical significance (Tables 2, 3, 4, and 5).

**Table 3:** Two-year follow-up: comparison of neurological findings among participants pre vs. post-surgery

	Normal before surgery^a^	Normal after surgery	Improvement after surgery^b^	No change in abnormal finding^b^	Worsening after surgery^c^
Strength					
Deltoids	15/19 (78.9%)	18/19 (94.7%)	4/4 (100%)	0	1/15 (6.7%)
Biceps	15/19 (78.9%)	18/19 (94.7%)	4/4 (100%)	0	1/15 (6.7%)
Triceps	12/19 (63.2%)	17/19 (89.5%)	7/7 (100%)	0	2/12 (16.7%)
Grips	13/19 (68.4%)	17/19 (89.5%)	6/6 (100%)	0	2/13 (15.4%)
Quads	11/19 (57.9%)	16/19 (84.2%)	7/8 (87.5%)	1/8 (12.5%)	2/11 (18.2%)
Hamstrings	12/19 (63.2%)	15/19 (78.9%)	6/7 (85.7%)	1/7 (14.3%)	2/12 (16.7%)
Iliopsoas	10/19 (52.6%)	16/19 (84.2%)	9/9 (100%)	0	2/10 (20.0%)
Reflexes					
Biceps	12/18 (66.7%)	14/18 (77.8%)	4/6 (66.7%)	2/6 (33.3%)	2/12 (16.7%)
Triceps	13/18 (72.2%)	12/18 (66.7%)	2/5 (40.0%)	3/5 (60.0%)	3/13 (23.1%)
Patella	10/18 (55.5%)	12/18 (66.7%)	5/8 (62.5%)	3/8 (37.5%)	3/10 (30.0%)
Achilles	12/18 (66.7%)	12/18 (66.7%)	4/6 (66.7%)	2/6 (33.3%)	3/12 (25.0%)
Other					
Heel to toe	10/15 (66.7%)	13/15 (86.7%)	4/5 (80.0%)	1/5 (20.0%)	1/10 (10.0%)
Finger to nose	14/14 (100.0%)	14/14 (100.0%)	NA	NA	0
Rapid alternating movements	13/13 (100.0%)	12/13 (92.3%)	NA	NA	1/13 (7.7%)
Romberg	13/16 (81.3%)	14/16 (87.5%)	2/3 (66.7%)	1/3 (33.3%)	1/13 (7.7%)
Sensation to vibration	10/10 (100%)	10/10 (100%)	0	NA	0
Sensation to pinprick	7/16 (43.8%)	9/16 (56.25%)	5/9 (55.6%)	4/9 (44.4%)	3/7 (42.9%)
Absence of tremor	19/19 (100%)	16/19 (84.2%)	NA	NA	3/19 (15.8%)

^a^Some participants did not have completed documentation for certain pre-op findings

^b^Participants who had abnormal finding prior to surgery

^c^Participants who had normal finding prior to surgery and developed abnormal findings s/p surgery

**Table 4:** Two-year follow-up: comparison of CXA, Grabb, Mapstone Oakes and horizontal Harris measurements pre vs. post-surgery

Patient	CXA pre-op^a^	CXA post-op	Grabb-Oakes pre-op^b^	Grabb-Oakes post-op	HHM pre-op^c^	HHM post-op^c^
1	131	150	8	6		
2	135	151	7.5	8		
3	130	146				0.1
4	131	141	8.5	7.4		1
5	115	143	12	5.2	9.2	0.1
6	124	142	12	7.4		1
7	120	152	8.8	5	9.1	1
8	128	146	10	7.7		
9	130	149	10	8	4.3	
10	124	149	9.9	5.4		1
11	132	143	8	7.4	3.2	1.4
12	128	156	7.6	4.6		2
13	130	162				0
14	130	146	9	6.6	2.9	2
15	115	150	8.8	7		0
16	130	146	7.9	6.7		1
17	131	152	7.9	6	0.4	
18	128	140	9.6	7		
19	126	146	9.5	7.6		1
20	131	143	9.5	7.1		1

^a^Clivo-axial angle abnormal (≤ 135); abnormal preop 20/20; post-op 0/20

^b^Grabb-Oakes abnormal > 9, *n* = 9/18

^c^Horizontal Harris measurement: a difference of greater than 2 mm between flexion and extension is an abnormal translation. Abnormal *n* = 5/6 (pre-op), *n* = 0/14 (post-op)

**Table 5:** Five-year post-op presence and change in frequency of statistically significant symptoms/problems among participants (*n* = 20)

Symptom/problem	% Pre-surgery	% Post-surgery	% With improvement in frequency post-surgery*	% With worsening of frequency post-surgery^a^	*p* value^b^	% With onset post-surgery^c^
*Headaches*	100%	100%	65% (13/20)	0	*< 0.0001*	0
*Dizziness*	100%	85%(17/20)	75%(15/20)	15%(3/20)	*< 0.02*	0
*Balance problems*	85% (17/20)	75%(15/20)	58.8%(10/17)	11.8%(2/17)	*< 0.008*	0
*Memory problems*	80% (16/20)	80%(16/20)	25%(4/16)	12.5%(2/16)	*NS (< .1)*	0
*Walking problems*	80% (16/20)	50% (10/20)	68.8% (11/16)	6.3%(1/16)	*< 0.003*	25%(1/4)
*Vertigo*	65% (13/20)	55%(11/20)	84.6%(11/13)	0	*NS(.074)*	28.6%(2/7)
*Speech problems*	60% (12/20)	35%(7/20)	66.7% (8/12)	0	*< 0.006*	0
*Frequent daytime urination (>every 2 h)*	60% (12/20)	35%(7/20)	66.7% (8/12)	0	*< 0.04*	25%(2/8)

^a^For those participants who had presence of symptom/problem prior to surgery

^b^Comparing frequencies of symptom/problem pre vs. post-surgery, a significant *p* value indicates less frequent symptom/problem post-surgery

^c^For those participants who did not have the presence of symptom/problem prior to surgery

On neurological examination, those who were weak before surgery improved, though not completely. The ability to walk heel-to-toe, Romberg, and sensation were all improved. There was no significant improvement in reflexes (Table 3).

### Functional outcome

Function and the ability to return to work, as assessed with the Karnofsky Performance Scale, demonstrated a highly statically significant improvement (*p <* 0.001). Preoperatively, 12/20 subjects were completely disabled, and 4/20 were able to care for themselves only, but unable to go to work or school. Postoperatively, 3/20 showed no change and 3/20 worsened on the Karnofsky scale. However, 14/20 subjects improved in their Karnofsky score: 5/20 had improved in work/school status, and an additional two subjects were seeking part time work or about to begin school, for a total of 7/20. Many patients were able to return to caring for their families and enjoying life to some extent; overall, 10/20 had a Karnofsky of 80 or higher (Fig. 7**,** Table 6).

**Table 6:** Two- and five-year patient Karnofsky scores and current functioning levels

Patient #	Age at surgery	Gender	Karnofsky pre-op	Karnofsky 2 years post-op	Karnofsky 5 years post-op	Current work/school status	Present illnesses/contributing factors
1	18	F	50	80	80	In school, doing research	2015 hardware removal and fusion augmentation
2	11	F	30	50	70	Online school, part time	EDS issues-chronic pain, difficulty walking (WC for long dist), t-spine pinching, l-spine popping and sliding, both spasming, further surgical procedures between 2012 and 2014 including untethering of spinal cord, ACDF C3-C5 and C5-C6, LP and hardware revision C2-C3
3	17	F	80	90	90	In school/ waitressing	MVA 4–5 months ago with C7 fx, shoulder pain
4	17	F	40	80	60	Not in school	EDS issues, intracranial HTN, GI issues, recently failed lumbar shunt, (score goes to 80 when shunt is working), Had previous Chiai Decompression and duroplasty in 8/10 and 5/11; 4 shunt revisions 2013–2015, Also had ACDF C4-C5 and fusion T6-T11 in 2015; fusion revision T8-L4 12/6/16, fusion C2-T1 12/27/16, had immune reaction to bone dust with vascular swelling, volunteers at mom’s school when able.
5	20	F	50	80	70	Not in school or working	Had decompression in 5/09; EDS issues-dystonias/ dislocations, fatigue, pain all over/ back and leg pain, more dislocations and subluxations, slipped disk in back ~ 5×/week, fiancé helps with shopping, driving > 30 min, can do most ADL’s needs help with heavier tasks
6	20	F	50	40	40	Fully disabled/bed bound	EDS issues- severe dislocations, inc. ICP, cervical medullary syndrome, POTS, dysautonomias, J-tube, gastroparesis, clotting disorder, 5 clots incl. R internal jugular, migraines, intractable aura, GERD, constipation, dec. cog function, Patient had tethered cord procedure 11/2011, has moderate cognitive impairment, in house nursing/palliative care
7	43	F	60	70	80	Part time job	Fatigue, pain, arms/leg joints- is able to care for son
8	34	F	50	50	70	disabled	Had 2004 suboccipital decompression and previous TC procedure, EDS issues- IIH, Adrenal insuf, OA, MCAD, scoliosis, interstitial cystitis, 2013 tethered cord procedure, had hardware removal with augmentation of fusion 2015 with improved POTS, headaches are better but continue to keep her from working, she believes they may be related to IIH
9	17	F	60	80	90	Full time job	Still has blackout/dysautonomia issues and severe pain 1-2x yearly, had 2014 hardware revision, routine PT helps her function at higher level
10	28	F	50	60	70	disabled	Pituitary adenoma, failed hip surgery, possible eagle syndrome, tremors, adrenal insufficiency, acromegaly, daily H/A, dislocations, 4/14 hardware removal/fusion augmentation; fusion C2-T1 arthrex ligament augment 1/10/17, can do self care
11	18	F	80	60	90	Full time Student	Symptoms improved after stopping diazepam, had 2014 hardware removal with augmentation fusion, credits surgery and PT/life balance
12	17	F	30	90	80	Working part time	Headaches, joint pain elbows, knees, urinary problems, had MVA in July now 22 weeks pregnant (2/22), can do house work and self care
13	12	F	80	90	90	Student	Getting straight A’s, taking dance classes. Has 3–4 classes a semester, has found that school plus work is too much in that it increases fatigue/headaches and other symptoms
14	36	F	60	70	70	Disabled	Pressure, LP shunt placement 2013, revision 10/15, tethered cord procedure in 2012, hardware revision in 2013, ACDF C3-C4 2014, fusion C4-T1 2015. PANDAS, POTS, ICH, June surgery/does self care but needs help with heavier chores, shopping, driving more than locally-no highway driving, has headache, instability, shoulder blade pain, 10/16 fusion C2-T1
15	19	M	70	90	80	Student, part time	Pain and neuropsych symptoms, 2010 TC and LP shunt Takin 1 class at community college, lives at home
16	18	F	80	80	90	Working from home/part time work	Had Chiari decompression procedures 3x in 2010,2012 hardware revision and 2015 fusion augment/ chronic H/A thinks she will need pain management for full time work, 3/7/17 TC
17	26	F	50	50	70	Disabled	Pain and fatigue from EDS, unrelated to surgeries, can function ~ 90–120 min QD, dysautonomias, hypothyroidism, Raynauds, on ssdi, uses adaptive equipment
18	31	F	60	50	50	Disabled	Had ACDF C5-C6 6/2016, before that had severe H/A, trouble walking, joint pain, hip problems, neck pain further down upper back/arms/shoulders. Needs assistance with prepping food and bathing
19	53	F	60	90	80	Retired, thinks she could work part time otherwise	Superior mesenteric artery syndrome “nutcracker syndrome”, L renal vein compression, pneumonia, vascular digestive issues, had SMA transposition (1–2 yr. recovery), was posted for hardware revision with augment fusion occ-c1/c2 in April 17, surgery has not happened as of May 17. Does self-care
20	17	F	60	80	60	New born, unable to work	Had a decompression in 10/07, TC in 8/09; EDS issues- Lumbar shunt,, inc. ICP, c-spine pain, PT helps, 27 wks pregnant as of 9/16- needs help with different ADLs based on pain/energy, has lumbar shunt pressure issues that have to wait to be addressed postnatally, walks ~ 20 min, WC after that, PT helps, 2014 LP and hardware removal, augmentation of fusion, has 10wk old as of 2/22, needs help with basic housework

Karnofsky scores were reassessed post-operatively at 5 years. There remained statistically significant improvement (*p <* 0.003). Eleven of 20 patients remained in work or school; 17/20 had improvement in Karnofsky compared to pre-op, 1/20 had no change and 2/20 had worsened (Fig. 7).

There was no significant difference found between the 2-year and 5-year Karnofsky (*p <* 0.43) **(**Fig. 7**)**. Compared to the 2-year score, the 5-year post-op Karnofsky evaluation had improved in 8/20, showed no change in 6/20, and worsened in 6/20.

### Radiological outcomes

Open reduction was successful in normalizing the CXA in every subject. Preoperatively, radiological examination demonstrated abnormal CXA (less than or equal to 135°) in 20/20 subjects, with an average CXA of 127° (Fig. 8). Post-operatively at 2 years, the average CXA was 148° (*p <* 0.001).

Preoperatively, the Grabb, Mapstone, Oakes measurement was made in 18 subjects; the methodology yielded a measurement greater than 9 mm in 9/18 subjects, constituting a high-risk category for ventral brainstem compression [7]. Postoperatively at 2 years, all subjects (20/20) were within the normal range (less than 9 mm).

Preoperatively, the horizontal Harris measurement demonstrated craniocervical instability in 5/6 patients; in these patients, there was pathological translation varying from a mean of 4 to 9 mm. Translation in the Harris measurement was the difference between that measured on flexion and that measured on extension in the upright MRI [76–78]. Post-operatively, as a consequence of the reduction and stabilization, the translation by horizontal Harris measurement was less than or equal to 1 mm in 12 out of 14 subjects and equal to 2 mm in 2 out of 14 subjects (Table 4).

Eleven of twenty had Chiari malformation (descent of the cerebellar tonsils of 5 mm or more below McRae’s Line), of whom, five had undergone a prior suboccipital decompression; one had a Chiari Zero; six subjects had low-lying cerebellar tonsils (cerebellar ectopia, where the descent of the cerebellar tonsils did not reach the 5 mm threshold).

The fusion rate as determined by postoperative CT scan was 100%.

### Complications of surgery

There were no deaths or major peri-operative morbidities. Two subjects underwent transfusion intraoperatively. Two subjects had superficial infections, of which one returned to the operating room for closure of the rib wound dehiscence. Mild to moderate pain (3/10) at the rib harvest site was common at 2 years, substantially abating at 5 years.

Despite the loss of 20 to 30° of flexion and extension at the craniocervical junction, and 35° of rotation to each side at C1–C2, range of motion was not a concern for any of these subjects. One to four years after the craniocervical fusion, some subjects developed pain over the suboccipital instrumentation (the “screw saddles”) due to tissue thinning, and requested hardware removal (8/20 subjects).

## Discussion

This is the first 5-year study to retrospectively examine the outcome of craniocervical fusion in patients with a hereditary connective tissue disorder and craniovertebral instability. The study reviews responses of a cohort of 20 subjects disabled with pain and neurologic deficit, who had failed non-operative regimens, who presented with kyphotic clivo-axial angle (CXA less than 135°) and basilar invagination, or instability at the craniocervical junction (CCI) in the setting of a hereditary connective tissue disorder, such as Ehlers-Danlos syndrome. Eighteen of the twenty subjects had low-lying cerebellar tonsils, including Chiari malformation, type I or type 0.

### Ehlers-Danlos syndrome

Emblematic of the approximately 50 hereditary connective tissue disorders are the Ehlers-Danlos syndromes (EDS), a heterogeneous group of heritable, connective tissue disorders characterized by joint hypermobility, skin extensibility, and tissue fragility. The 2017 classification [84] recognizes 13 subtypes, which for the most part are due to mutation of genes that encode fibrillary collagens or the enzymes involved in post-translational modification of collagen. Hypermobile type EDS (h-EDS) is diagnosed on the basis of clinical findings [85], while molecular testing is available to confirm most other forms of EDS [84, 86–88]. The neurological and spinal manifestations of h-EDS and the classic form of EDS have been reviewed [41, 89, 90].

### Ligamentous laxity at the craniocervical junction

EDS is fundamentally a disorder of collagen and other structural components of connective tissue, characterized by incompetent ligaments, joints, and spine. Ligaments are the major occiput–C1 stabilizing structures [4]. In the presence of ligamentous laxity or disruption, the CCJ is incompetent in the execution of multiaxial movements [91, 92]. Craniocervical instability (CCI) is thus a manifestation of ligamentous laxity in EDS [18, 53, 61, 62, 93, 94].

Most atlanto-occipital joint movement occurs in flexion-extension, and axial rotation is normally limited; greater than 5° rotation at the occipito-atlantal joint is abnormal [95]. The lateral atlanto-occipital ligament prevents excess rotation between occiput and atlas; incompetence of the lateral atlanto-occipito ligament results in increased contralateral rotation by 3 to 5°. The tectorial membrane and nuchal ligament, composed parallel bundles of collagen, restrict hyperflexion, maintain posture, and help to restore normal position [96]. In the population of patients with hypermobility connective tissue disorders, incompetent ligamentous connections from the skull to the spine may progress to CCI.

### Neurological deficit has been attributed to ligamentous laxity at the craniocervical junction

Neurological injury is common in many other connective tissue disorders, such as rheumatoid arthritis, Down syndrome, and hereditary disorders such asosteogenesis imperfecta [10, 17, 18, 21, 25–28, 30–35, 37, 42, 44–46, 48, 49]. Non-disruptive stretch injury of the neuroaxis has been attributed to hypermobility of the craniocervical junction in infants and children, in whom the axonal lesions tend to be localized to the dorsal brainstem, lower medulla, in particular the corticospinal tracts at craniocervical junction [97]. Similar histopathological findings of nerve injury were seen in the lower brainstem and spinal cord, in adults [30, 71, 98–100].

In the EDS population, motor delay, developmental coordination disorder, headaches secondary to spinal compression, clumsiness, and the relatively high rate of dyslexia and dyspraxia have been recognized as a consequence of the effects of ligamentous laxity upon the central nervous system [18, 51, 53–60, 62, 66]. Interdigitation of the posterior-atlanto-occipital membrane with the pain-sensitive spinal dural layer has also been implicated in the genesis of headache [101].

### The cervical medullary syndrome

The cervical medullary syndrome, also known as “craniocervical syndrome” (ICD-9-CM Diagnosis Code 723.2; ICD-10-CM Diagnosis Code M53.0), comprises those symptoms commonly attributed to lower brainstem and upper cervical spinal cord pathology, usually in the presence of a “complex Chiari” (Chiari malformation with basilar invagination or craniocervical instability) [1, 3–5, 77, 79].

In the present study, all subjects presented with headache, fatigue and dizziness, and most reported, in descending order of frequency: weakness, neck pain, imbalance, night awakenings, memory difficulties, walking problems, sensory changes, visual problems, vertigo, altered hearing, speech impediments, micturition issues and dysphagia, and syncopal episodes. In aggregate, these symptoms are reasonably described as the “Cervical Medullary Syndrome” [1, 77].

While there is an overlap of clinical findings, the clinical presentation of the pure Chiari malformation differs from the complex Chiari malformation. Chiari I malformations are characterized primarily by the suboccipital “cough headache” exacerbated by Valsalva, cough or straining-dizziness, elements of cerebellar dysfunction, lower cranial nerve deficits, and gait problems [102]. On the other hand, the “Complex Chiari” with ventral brainstem compression or craniocervical instability present with other genetic conditions—such as HOX D3 homeotic transformation, Klippel Feil malformation, hereditary connective tissue disorders [102–104]—and is characterized by pyramidal changes, with weakness, hyperreflexia, pathological reflexes, paresthesias, and sphincter problems, in addition to headache, neck pain, dizziness, vertigo, dyspnea, dysphonia, altered vision and hearing, syncope, gait changes, and altered sleep architecture [5, 7, 10, 30, 70, 71, 105–107]. Dysautonomia has also been attributed to basilar impression [108].

### Radiological metrics in the diagnosis of basilar invagination and CCI

Three radiologic metrics used in this study, the Clivo-axial angle (CXA), the horizontal Harris Measurement [78], and the Grabb, Mapstone, Oakes measurement [7, 78] have been adopted as common data elements (CDEs) by the NIH/NINDS, and characterized useful in identifying possible CCI and basilar invagination [1, 76, 77]. The CXA of less than 135° is considered potentially pathological [10, 12, 18, 30, 70–75, 79, 109]. Salutary consequences have been attributed to the correction of the CXA [10, 12, 69, 70, 107].

The Grabb, Mapstone, Oakes measurement of 9 mmor more suggests high risk of ventral brainstem compression, requiring consideration for craniospinal reduction or transoral decompression, and fusion stabilization [7, 77, 79].

The horizontal Harris measurement (or BAI) was useful in demonstrating craniocervical instability. Normally, the basion pivots on a point above the odontoid, and there is no measurable translatory movement between flexion and extension. A change in the horizontal Harris measurement of 2 mm or more, as measured in flexion and extension images, represents pathological translation between the basion and odontoid [1, 10, 76, 77, 79, 110–114].

### Non-operative management of patients with craniocervical instability due to hereditary connective tissue disorder

Patients should be given a specific diagnosis to validate their concerns, and allay their fears. Rigorous instruction should follow to avoid aggravating activities—impact sports and prolonged sitting or driving, the importance of frequent rest periods, physical therapy—for strengthening, sagittal balance, posture and cardiorespiratory fitness, and judicious use of appropriate bracing, to be accompanied by isometric exercises. When possible, treatment of co-morbid conditions should be undertaken.

Craniocervical fusion should be considered the last option, to be engaged when non-operative treatment has failed.

### Indications for surgery

Posterior occipito-cervical fusion is indicated in patients who present with basilar invagination, instability or abnormal biomechanics, and cervical medullary syndrome [13, 21, 25, 112, 115].

Therefore, at the time of decompression of a Chiari malformation, the finding of basilar invagination or craniocervical instability should prompt consideration of fusion and stabilization [2, 3, 11, 18, 19, 21–23, 116, 117].

In this study, indications for surgery included disabling headache or neck pain, symptoms constituting the cervical medullary syndrome *with* demonstrable neurological findings, congruent radiological findings, a determination on the part of the patient that they were unable to continue in the normal activities of daily living, and failed non-operative treatment.

Headache should not be attributed a priori to craniocervical instability. In the hereditary connective tissue disorders, headache may have many origins: cervicogenic, vessel dissection, or venous occlusive disease or thrombosis, intracranial hypertension or hypotension, temporomandibular joint syndrome, inflammatory and infectious disorders, neuralgia and migrainous conditions, postural orthostatic tachycardia syndrome (POTS), or mast cell activation syndrome (MCAS) [41, 118, 119].

Radiological metrics are useful guidelines, but not indications, per se, for surgery. The radiological indications were congruent with the treatment algorithm previously established [70]. Abnormal radiological metrics may exist in patients with no neurological symptoms.

A number of subjects with CCI were also found to have atlantoaxial instability, a radiological and clinical finding that did add weight to the decision to proceed to surgery. Occipitocervical fusion is indicated in some circumstances for atlantoaxial instability alone, or for complex cervical deformities [21, 27].

A patient with hereditary disorder is at risk for multilevel instability issues; any injury or period of disability may result in exacerbation of instability [120]. The complexity of these patients warrants a rigorous selection process. Selection of candidates for surgery should follow standard guidelines and indications for instability, the diagnosis of which often requires dynamic imaging [13, 14, 70]. Occipitocervical fusion should be considered the last treatment option in this patient population [41].

### Surgical open reduction

The reduction should be executed in a thoughtful and deliberate manner to avoid incorrect or painful malalignment, “star gazing” from excessive extension or conversely a downward gaze. If the cranium is inadequately extended, the *oropharyngeal space* may be decreased, and the patient may exhibit severe dysphagia or potentially life-threatening dyspnea [121]. To maintain appropriate oropharyngeal space, the surgeons extended the cervical spine to maintain 2 cm between the anterior spinal line and the posterior edge of the mandible, as seen on lateral fluoroscopy. In most cases, the basion was translated posteriorly to lie above the midpoint of the odontoid. The kyphotic angulation of the brainstem over the odontoid process, as measured by the CXA, was normalized by extension of the cranium at the craniocervical junction, thereby decreasing the *fulcrum effect of the odontoid* [49]**,** and the mechanical stress on the brainstem [10, 12, 30, 41, 109, 122]. We attempted to achieve a mild cervical lordosis.

### Reduction, fusion/stabilization appears to improve pain and neurological deficit

There was 100% follow-up at 2-year and 5-year follow-up. Except for the neurological exam, the clinical data was collected by a third party, and de-identified. All patients were satisfied with the surgery, would repeat the surgery given the same circumstances, and reported improved quality of life. All but one patient would recommend the surgery to a family member. Eighteen of the twenty patients reported that the craniocervical fusion surgery decreased their limitations; two reported continued limitations from other medical problems and spinal instability elsewhere.

Postoperatively, at the 2-year follow-up, patients demonstrated a statistically significant improvement in in frequency and severity of headache, speech, memory, vertigo, dizziness, gait, balance, and urinary frequency. There were also improvements in most patients with tremors, syncope, imbalance, hearing problems, dysarthria, swallowing difficulty, numbness of the upper and lower extremities and back, neck pain and upper extremity weakness.

At 5 years, there remained statistically significant improvement in headaches, dizziness and imbalance, gait, speech problems, and frequent daytime urination**.** Though not statistically significant, there was also continued improvement in upper extremity, back and lower numbness, syncope, upper and lower extremity weakness, swallowing difficulty, and hearing problems.

At the 2-year period, the improvement of the Karnofsky performance score was statistically significant and remained significantly improved over the 5-year follow-up period, with the majority of subjects returning to employment, school, or work in the home. This improvement was supported by the observed improvement in neurological deficits; weakness, heel-to-toe walking, Romberg and sensation.

### Co-morbid conditions in this population that confounded the outcome

At 5 years, 8/20 patients reported disability from co-morbid conditions. In keeping with the literature, most patients presented with postural orthostatic tachycardia syndrome and other manifestations of dysautonomia; many patients received diagnoses of abnormalities of CSF hydrodynamics with intracranial hypertension or hypotension, abnormalities of intracranial venous drainage due to sinus stenosis or jugular vein stenosis. Migraine headaches and temporomandibular joint dysfunction were very common. A majority of patients had vitamin and trace element deficiencies. Many patients demonstrated cervical instability with cervicogenic headaches. Gastroparesis, superior mesenteric artery syndrome, mast cell activation syndrome occurred and endocrine disorders. Several patients were diagnosed with movement disorders, Tarlov cysts, kypho-scoliosis, tethered cord syndrome, neuromuscular disorders, anxiety, and depression [18, 41, 123–129].

A multi-disciplinary team, familiar with the many co-morbidities and the generalized ligament laxity throughout the spinal column, is necessary to address the many issues in order to improve the well-being of the patient with a hereditary connective tissue disorder.

### Complications of surgery

There were no deaths or major peri-operative morbidities. There were two patients who underwent transfusion intraoperatively, two with superficial infections of whom one returned to the operating room for closure of the rib wound dehiscence. Mild to moderate pain at the rib harvest site was common at 2 years, substantially abating at 5 years. Spinal instability is a potential complication of rib harvest, but was not reported in this group.

The absence of screw malposition and vertebral artery injury [29, 130] is attributed in part to improvement in instrumentation, preoperative CT to examine the anatomy, and intra-operative fluoro-CT to assess the construct real-time.

No patient complained of decreased neck range of motion after surgery. Despite the loss of approximately 20° to 30° of flexion and extension at the craniocervical junction, and 35° of rotation to each side at C1–C2, range of motion was not a concern for any of these patients.

One to four years after the craniocervical fusion, some patients developed pain over the suboccipital instrumentation (the “screw saddles”) due to tissue thinning, and requested hardware removal (8/20 subjects). The authors have, therefore, adopted lower profile craniocervical instrumentation. A smaller profile generally requires a smaller size and smoother outer contour of the instrumentation. The instrumentation should be configured to allow placement as low as possible over the cranium, to increase the thickness of the tissue overlying the instrumentation.

### Concerns about adjacent segment degeneration

The presence of premature disk degeneration and ligamentous laxity with excessive spinal range of motion that characterizes hereditary connective tissue disorders, makes this population vulnerable to both axial and appendicular joint pathology. In most, there had been some degree of instability in the mid-cervical levels before the craniocervical fusion, and many of these subsequently underwent further cervical spine surgery (Table 7). It was surprising, however, that the adjacent segment, C2–3, was rarely the site of isolated segment instability after the craniocervical fusion.

**Table 7:** Additional surgical procedures

	Before CVF	After CVF	*P* value
All surgeries	30% (6/30) range 0–3, avg. 0.5	60%(12/20) range 0–9, avg. 1.7	*<***0.04**
Chiari decompression	25% (5/20)^a^	0	*<***0.05**
TCR	15% (3/20)	25%(5/20)	NS
LP shunt	5% (1/20)	15% (3/20)	NS
Hardware removal with fusion augment	0	40%(8/20)	*<***0.002**
Fusion at other level	0	25% (5/20)	*<***0.04**
ACDF	0	20%(4/20)^a^	NS
C2-T1 fusion	0	15%(3/20)	NS
Thoracic Fusion	0	10%(2/20)	NS
Hardware revision, other level	0	10% (2/20)	NS
Shunt revision	0	10% (2/20)^a^	NS
Lumbar puncture	0	10% (2/20)	NS

^a^Patients with repeated procedures: one had two and another three Chiari decompressions, one had ACDF twice at different levels, one patient had shunt revision two and another four times. For fusion at other levels, two patients had three procedures, and another had two

Goel has suggested that ligamentous instability at the craniocervical junction decreases neuromuscular control, leading to further central nervous system injury in a reverberating process that is further exacerbated by the presence of malnutrition and loss of conditioning [120].

Therefore, the putative benefits of craniocervical fusion—improvement of neuromuscular control—should be weighed against the possibility of adjacent segment degeneration and increased proclivity to further spine surgery.

The many co-morbid conditions, the frequent osteopenia, and small bone structure of this population render the appearance of high surgical risk. Yet, surgical outcomes have been surprisingly gratifying, perhaps because this population is younger, and in some respects healthier than those in published studies of craniocervical fusion for rheumatoid arthritis, cancer, trauma, infection, and the elderly [21, 29, 32].

### Is kyphosis of the CXA a consideration in the determination to perform a fusion stabilization?

The clivo-axial angle (CXA) has a normal range of 145° to 165°. Flexion of the neck usually decreases the CXA by 10°, and extension of the neck increases the CXA by approximately 10°.

Nagashima reported an angle of less than 130° may produce brainstem compression [62, 73]. Van Gilder reported that a CXA of less than 150° was associated with neurological changes [67]. Kim, Rekate, Klopfenstein, and Sonntag reported that a kyphotic CXA (less than 135°) was a cause of failed Chiari decompression; subsequent open reduction to normalize the CXA resulted insubstantial improvement in 9/10 of the subjects, prompting the authors to describe the kyphotic CXA as a form of “non-traditional basilar invagination” [12]. Morishita suggested that a clivo-axial angle of less than 135° is a risk factor for spinal cord compression [131]. Kubota, in a retrospective series of foramen magnum decompression for Chiari and syringomyelia, reported that the syrinxes failed to abate in those patients in whom the CXA was less than 130° [15].

Brockmeyer, in a retrospective pediatric series of Chiari decompressions, reported that 20% of patients were returned to surgery for reduction and stabilization for kyphotic CXA, craniocervical instability, or the presence of a Chiari 1.5 [4], a finding echoed by Klekamp and others in the adult population [6, 7, 9, 13, 19, 21, 132, 133]. The medulla becomes kinked as the CXA becomes more kyphotic; increasing kyphosis of clivo-axial angle creates a fulcrum by which the odontoid deforms the brainstem [49, 132, 134]. A more complete treatise on the importance of the CXA has been presented elsewhere [70]. In this study, the mean preoperative CXA of 129° was increased to 148° by open reduction of the kyphosis, which correlated with patient improvement. However, the observed improvement may have been the result of craniocervical stabilization.

### Pathophysiology

A kyphotic CXA is associated with bending and strain of the lower brainstem and upper spinal cord, and a prelude to neurological deficit [9, 22, 32, 68, 135]. Stretching a neuron nerve decreases neural firing rate and amplitude [136]. The predominant substrate for deformity-induced injury is the axon: electron micrographs show clumping, loss of microtubules and neurofilaments, loss of axon transport and accumulations of axoplasmic material identified as *the retraction ball*, or *retraction bulb,* analogous to diffuse axonal injury (DAI) in the brain [137–142]. Axon retraction bulbs result from stretch/deformation injury and injury in animal models [98, 100, 143, 144], in the cortico-spinal tracts of the brainstem in infants with *shaken baby syndrome*, adults with spinal cord injuries, and in basilar invagination [30, 109, 143, 145, 146]. At the molecular level, stretching of nervous tissue deforms Na+ channels, causing increased membrane depolarization and a consequent deleterious influx of Ca++ [147]. The epigenetic effects of mechanical strain are manifest in the observation of increased expression of N-methyl-d-aspartate in the stretched neuron, altered mitochondrial function, and apoptosis [148–150].

The clinical improvement observed in this cohort is the presumed consequence of reduction of mechanical deformity of the nervous system and elimination or mitigation of microtrauma from craniocervical instability [10, 16, 49, 107], consistent with the experimental models of axons subjected to strain [149, 151–154].

### Controversy

Treatment of other forms of degenerative and hereditary connective tissue disorders is firmly established in the literature. However, treatment of the EDS patient has been problematic for several reasons. First, though EDS was first described in 1901, the recognition of spinal and neurological manifestations has been only recent [56, 62, 18, 41, 51, 53–55, 57–61, 66]. Because this information is new, there is a dearth of evidence upon which to base the management of these genetic disorders.

Second, EDS is considered an “invisible disorder.” EDS patients are characterized by youthful skin, and the appearance of good health, belying their severe pain and disability.

Third, the legion of disparate symptoms due to ligamentous weakness of the joints and spine, and the many co-morbid conditions that accompany EDS, result, understandably, in dismissal by healthcare providers because of the large number of seemingly disparate symptoms.

The authors advocate that the indications for craniocervical fusion should be no different than for the non-EDS population, with the caveat that conditions of ligamentous laxity often require dynamic imaging to demonstrate the pathology [1, 13, 14, 27, 77, 112, 155]. Occiput to C2 bone fusion, as opposed to atlantoaxial fusion in conjunction with fixation, has been discussed [3, 38, 69, 82, 115, 155]. A comparison of various methodologies for bone fusion has also been discussed [83].

### The economic significance of hypermobility connective tissue disorders

Treatment of the EDS population is problematic because of the diverse *spectrum* of disease severity and presentation for whom, in the majority of cases, there is no genetic testing available. In the experience and belief of most EDS care providers, EDS patients suffer through scores of visits to specialists over a mean of 10 years before the diagnosis of EDS is made, during which time they consume vast medical resources through emergency room visits, and unscheduled, often prolonged, admissions to hospital.

The epidemiology of EDS is not known; however, there is little phenotypic difference between patients with h-EDS and the very large population previously diagnosed with joint hypermobility disorder (now referred to as hypermobility spectrum disorder) sharing the same early degeneration of the spine and joints, and the same co-morbid conditions [156–158]. Therefore, the authors believe that earlier recognition of these hereditary disorders would substantially reduce costly specialty visits, improve care of this patient population. Early recognition, prudent management, and non-operative therapy may be adequate to stabilize the patient and obviate need of surgery in many cases.

In this cohort, 55% of subjects have returned to work and are paying taxes, or attending school full or part-time with the prospect of future employment, or serving society through caring for their families.

### Limitations of the study

This IRB study is a single-center, non-controlled analysis of a small cohort of subjects, referred by medical providers from a broad geographical area (USA and Canada). The study was conceived prior to any surgery, but the subjects were not enrolled until after surgery. Therefore, this should be considered a retrospective study. The outcomes data is to some extent obfuscated by the presence of previous Chiari surgery (five), multiple co-morbid conditions common to EDS, and multiple surgeries within the 5-year follow-up period (12/20). The complexity of the co-morbid conditions and other surgeries are prohibitive to more complex statistical methods. These patients appeared to be the most seriously affected patients within the spectrum of hereditary connective tissue disorders.

Not every patient had cerebellar ectopia (18/20). Subjects may have inaccurately reported the severity of their preoperative symptoms upon questioning at the 2-year follow-up and may have exaggerated the degree of improvement. However, accuracy of reporting was improved through the employment of two independent researchers, who performed the subjects’ interviews at 2 and 5 years. Some subjects may have seen surgery as means validation of their suffering. There was no control for a placebo effect [159].

## Conclusion

This study supports the hypothesis that craniocervical reduction, stabilization, and fusion are feasible and associated with clinical improvement in patients in the HCTD population with Chiari malformation or cerebellar ectopia, kyphotic clivo-axial angle, ventral brainstem compression, and/or craniocervical instability. The neurological and functional improvements associated with craniocervical fusion/stabilization appear to be clinically significant and durable. That said, craniocervical fusion should be considered as a last resort after a reasonable course of non-operative treatments.
